# Validated Stability-Indicating GC-MS Method for Characterization of Forced Degradation Products of *Trans*-Caffeic Acid and *Trans*-Ferulic Acid

**DOI:** 10.3390/molecules26092475

**Published:** 2021-04-23

**Authors:** Maša Islamčević Razboršek, Milena Ivanović, Mitja Kolar

**Affiliations:** 1 Faculty of Chemistry and Chemical Engineering, University of Maribor, Smetanova Ulica 17, SI-2000 Maribor, Slovenia; masa.islamcevic@um.si (M.I.R.); milena.ivanovic@um.si (M.I.); 2 Faculty of Chemistry and Chemical Technology, University of Ljubljana, Večna Pot 113, SI-1000 Ljubljana, Slovenia

**Keywords:** caffeic acid, ferulic acid, stability studies, GC-MS, forced degradation

## Abstract

When dealing with simple phenols such as caffeic acid (CA) and ferulic acid (FA), found in a variety of plants, it is very important to have control over the most important factors that accelerate their degradation reactions. This is the first report in which the stabilities of these two compounds have been systematically tested by exposure to various different factors. Forced degradation studies were performed on pure standards (*trans*-CA and *trans*-FA), dissolved in different solvents and exposed to different oxidative, photolytic and thermal stress conditions. Additionally, a rapid, sensitive, and selective stability-indicating gas chromatographic-mass spectrometric method was developed and validated for determination of *trans*-CA and *trans*-FA in the presence of their degradation products. *Cis*-CA and *cis*-FA were confirmed as the only degradation products in all the experiments performed. All the compounds were perfectly separated by gas chromatography (GC) and identified using mass spectrometry (MS), a method that additionally elucidated their structures. In general, more protic solvents, higher temperatures, UV radiation and longer storage times led to more significant degradation (isomerization) of both *trans*-isomers. The most progressive isomerization of both compounds (up to 43%) was observed when the polar solutions were exposed to daylight at room temperature for 1 month. The method was validated for linearity, precision as repeatability, limit of detection (LOD) and limit of quantitation (LOQ). The method was confirmed as linear over tested concentration ranges from 1−100 mg L^−1^ (r^2^s were above 0.999). The LOD and LOQ for *trans*-FA were 0.15 mg L^−1^ and 0.50 mg L^−1^, respectively. The LOD and LOQ for *trans*-CA were 0.23 mg L^−1^ and 0.77 mg L^−1^, respectively.

## 1. Introduction

Many publications have investigated phenolic compounds—constituents of various plants—in terms of their role as natural antioxidants. It is well known that phenolic compounds have many more bioactive applications, such as anti-inflammatory, anti-tumor, antimicrobial and obesity-inhibiting functions, and have therefore, received a great deal of attention from scientists in various research areas over the past decades [1]. Consequently, natural plant extracts enriched with different classes of phenolic compounds (flavonoids, anthocyanins, phenolic acids, etc.) have generated a great deal of interest and there is a strong demand for such extracts today. However, for the practical application of these extracts in various industries (food, pharmaceuticals, cosmetics, etc.), appropriate stability studies are often required. Indeed, when working with compounds that are prone to isomerization, decomposition, or the formation of racemic mixtures, they should be handled with care and strict precautions should be taken with respect to illumination, the use of protic organic solvents, elevated temperatures, and long-term analysis in order to minimize their isomerization/decomposition. Isomerism is important in the field of pharmacology as it is known that isomers can differ in their pharmacokinetic and pharmacodynamic properties [2]. However, despite the abundance of articles related to phenolics, only a few of them mention their instability during extraction or analytical procedures [3,4,5,6,7,8,9,10,11].

Phenolic acids (PAs) are precursors for the biosynthesis of many other phenolic compounds, such as flavonoids, lignin, chalconoids, etc. Due to their antioxidant and prooxidant properties and their beneficial effects on human health, PAs represent a significant part of the human diet [12,13,14].

Among the PAs, (E)-3-(3,4-dihydroxyphenyl) prop-2-enoic acid (caffeic acid-CA) and (E)-3-(4-hydroxy-3-methoxy-phenyl) prop-2-enoic acid (ferulic acid-FA) belong to the group of hydroxycinnamic acids with pronounced bioactivity and are important not only for medicine and pharmacy, but also for the food and cosmetic industries [15,16]. In nature, hydroxycinnamic acids are often present as *trans*-isomers, but after exposure to various extreme conditions (UV radiation, high temperatures, strong daylight, etc.), a gradual formation of *cis*-isomers may occur [7].

**Figure 1:**
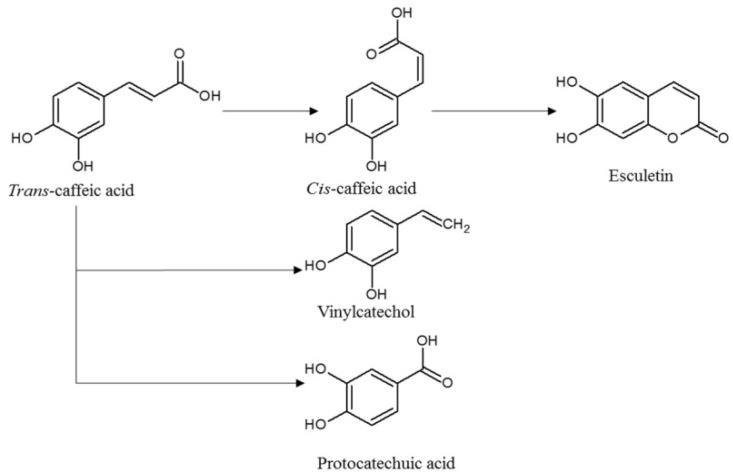
Proposed mechanism for the photodegradation of *trans*-CA [6].

CA has been found in high concentrations in coffee, propolis [17] and herbs of the *Lamiaceae* family [18]. It can be present in the form of amides, esters or glycosides either as a monomer, dimer or trimmer. In plants, CA is often found in combination with its most well-known dimer, rosmarinic acid [18]. It has been reported that CA has a wide range of potential pharmacological effects in both in vitro research and animal models, including an inhibitory effect on cancer cell proliferation [19,20]. Several studies have confirmed that *trans*-CA is not stable under the influence of light and that isomerization from the *trans*- to the *cis*-form as well as photoconversion to esculetin (6,7-dihydroxy-2H-1-benzopyran-2-one) can occur [4,6,10]. The proposed mechanism of the photo-degradation of *trans*-CA is shown in Figure 1.

FA, like many other natural phenolic compounds, is an in vitro antioxidant [21] and has been identified as a potential therapeutic agent for a variety of diseases, including neurodegenerative diseases, cancer, diabetes, cardiovascular dysfunction, inflammatory diseases, Alzheimer’s disease and also aging [22,23]. It has also been demonstrated that *trans*-FA is unstable under various conditions [24]. However, antioxidant studies conducted on the *trans*- and *cis*-isomers of FA, isolated from rice bran, showed that the *cis*-isomers exhibited almost identical DPPH hydroxyl radical and ABTS^+^ radical scavenging activities as the corresponding *trans*-isomers [9,11].

Many analytical techniques have been developed and validated for the quantitative determination of CA and FA from various natural sources, including voltammetry [25,26], UV-VIS spectrophotometry [27], near-infrared spectrophotometry (NIRS) [28], and chemiluminescence [29]. However, the literature indicates that the most common technique for this purpose is high-performance liquid chromatography with diode-array or ultraviolet-visible detection (HPLC-DAD or HPLC-UV/ViS) [30,31,32]. The determination of natural antioxidants by HPLC is a rapid and simple technique, but also has some limitations, especially in terms of sensitivity and the matrix effect [33]. A minor disadvantage of the GC-MS method compared to HPLC is the need for a derivatization step, which is necessary to ensure the volatility and thermal stability of the compounds [33]. However, GC-MS also offers a number of advantages, such as the simultaneous, complete and high-resolution separation of geometric isomers in a single chromatographic run, its sensitive detection and its unambiguous identification and quantification [34,35,36].

The GC-MS method can be used for studies that aim to determine the optimal conditions for efficient extraction, isolation, and quantification of PAs from different materials. For example, the standardization of propolis extracts and the development of extraction procedures and analytical methods for the classification of propolis series material or propolis-based pharmaceutical products still poses a great analytical challenge due to the chemical diversity of the ingredients. In this sense, the identification of characteristic marker compounds for the best profiling and classification (botanical classification, classification of origin, etc.) of propolis samples is a common procedure [37,38]. The desired classification is often based on the content of phenolic components, especially phenolic acids [37,39,40]. Consequently, the need for their quantitative determination and statistically acceptable results is obvious. Similar requirements were found for the chemometric classification of red wine samples [34].

Although few studies have been reported on the *trans-cis* photo-isomerization of investigated PAs [3,5,6,8], this is the first study in which the stability of *trans*-CA and *trans*-FA in two organic solvents (methanol and tetrahydrofuran) have been systematically tested under a wide range of stress conditions. The results of this study have the potential to be very useful for researchers focusing on studying target PAs, in the sense that they can be used to prepare and analyse them avoiding their isomerization/degradation, and consequently, inaccurate results.

## 2. Results and Discussion

CA and FA belong to the group of pharmacotherapeutics and are frequently used as ingredients in drugs, cosmetic products and dietary supplements. Therefore, it is very important to measure the degree of isomerization and to determine the individual isomers. In general, any setup that provides sufficient energy to the compound can isomerize it. This can be done by heating, irradiation with visible or UV light, or by using a catalyst. Excitation of electrons from the π to the π∗ (bonding orbital to anti-bonding orbital) allows free rotation around the σ bond. Relaxation back to the π orbital leads to a different configuration [41]. Cinnamic acids, such as CA and FA, are particularly susceptible to this type of reaction. At this stage, the GC-MS method may be the preferred choice to determine isomers. Namely, a good resolution of all isomers can be achieved on a single column, and although mass spectrometers cannot always distinguish between isomeric structures, they can be easily distinguished with different retention times [36].

In this work, the stability studies of CA and FA under the different stress conditions were systematically evaluated with the optimized GC-MS method.

### 2.1. Method Validation

The developed GC-MS method was partially validated for linearity, precision (as intraday and interday repeatability), LOD and LOQ. Linear regression analysis proved that the responses for the compounds studied were linear over the concentration ranges tested, from 1 to 100 mg L^−1^.

The correlation coefficients (*r^2^*) were above 0.999 for both compounds in all calibration curves. The repeatability of chromatographic analyses was confirmed by RSD, and this varied from 2.7% to 5.4% for FA, and from 1% to 5.7% for CA. The LOD and LOQ for *trans*-CA were 0.23 mg L^−1^ and 0.77 mg L^−1^, respectively. The LOD and LOQ for *trans*-FA were 0.15 mg L^−1^ and 0.50 mg L^−1^, respectively.

The obtained values of sensitivity and repeatability of the proposed method fully satisfy the set requirements of the study. Although slightly lower LOD and LOQ values were obtained in the study published by Lara-Guzmàn et al. [42], a direct comparison of the results is not possible, since in our case, the quantification was performed in the SCAN mode, while the SIM mode was used in the case of the previously published study [42].

### 2.2. Stability of Trans-Caffeic Acid

Although water and ethanol (EtOH) are the most commonly used solvents in the cosmetic, food, and pharmaceutical industries, authors have often highlighted MeOH as the best solvent for the quantitative extraction of bioactive components, such as phenolic acids, from various natural sources [43,44]. Our previous studies have shown that the isomerization of phenolic acids (e.g., rosmarinic acid) was carried out to a greater extent in protic solvents such as MeOH, but not in THF as an aprotic solvent [36]. Therefore, the aim of this study was to investigate the stability of the two most abundant phenolic acids (CA and FA) in these solvents. The storage stability of the selected compounds was monitored in accordance with the literature [36], exposed to different conditions over 60 days: (a) in the freezer at −18 °C; (b) under room temperature and darkness; (c) under room conditions at sunlight.

From the results presented in Table 1, it is evident that the content of *Cis*-CAin freshly prepared standard solutions was statistically insignificant (between 1% and 3%). A typical chromatogram of silylated compounds in a freshly prepared solution of *trans*-CA in MeOH is shown in Figure S1 (Supplementary Material). After one day of storage in the refrigerator at −18 °C or at room temperature (RT) in darkness, the *trans*-CA dissolved in the two solvents (MeOH or THF) was found to be quite stable, and isomerization occurred only in a small proportion (from 3.2% to 5.1%) (Table 1). As expected, a much more progressive isomerization from *trans*- to *cis*-CA was observed when the solutions were exposed to RT and daylight for 1 day (up to 28%). For comparison, the study published by Yáñez et al. showed that the influence of sunlight had a significant effect on the catalytic degradation of *trans*- CA from wine industry wastewater by TiO_2_ [45]. Morever, the results of our study showed that the decrease in the amount of *trans*-CA dissolved either in MeOH or in THF at different storage conditions coincided with the increase in the amount of *cis*-CA. In general, when *trans*-CA was dissolved in THF or MeOH and stored in darkness or in a refrigerator at −18 °C, most of the isomerization from the *trans*- to the *cis*- form occurred between day 1 and day 10, but then a slight increase in favor of the *trans*- isomer was observed between day 10 and day 20. However, after day 20, a decrease in the *trans*- isomer was again noted. This phenomenon can be explained by the fact that isomerization is a reversible reaction, in which *cis*-cinnamic acids or some other *cis*-compounds can spontaneously convert back to *trans*-isomers and vice versa [46,47]. Exposure to more protic solvents, higher temperatures and longer time periods led to higher isomerization/degradation. *Cis*-CA was the only degradation product of *trans*-CA under all tested conditions, confirmed by GC-MS. Further isomerization of *cis*-CA to esculetin [6,8] and other degradation products was not observed in our study. This result can be probably explained by the fact that the intramolecular cyclization of *trans*-CA to esculetin is a pH-dependent process [6,8]. Indeed, in the study published by Parrino et al. it was demonstrated that only in the presence of oxygen and at alkaline pH values (higher than 6) is the photoisomerization of *trans*-CA followed by intramolecular cyclization to esculetin [8]. The complete results of the stability tests for pure *trans*-CA dissolved in two different solvents are shown in Table 1.

#### Analysis and Identification of Caffeic Acid Isomers

**Figure 2:**
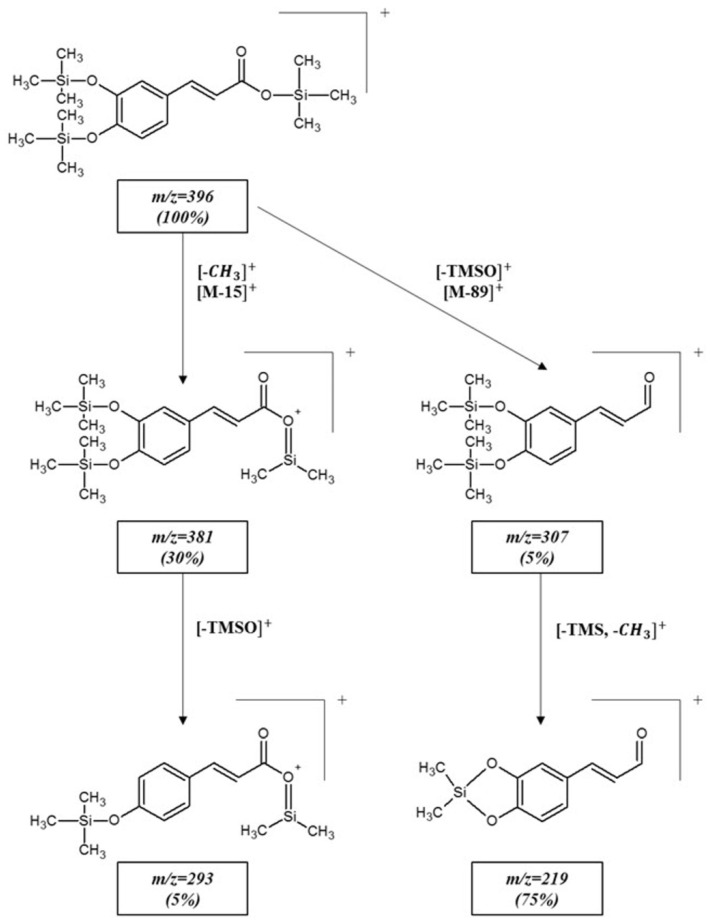
Proposed fragmentation pathway of the *trans*-CA-3-TMS derivative obtained under positive EI^+^ conditions.

The mass spectra of TMS-*trans*-CA and TMS-*cis*-CA (Figure S2, Supplementary Materials) are almost identical, but when comparing both spectra in detail, it can be seen that they differ slightly with respect to the intensities of the certain fragment ions. The most intensive (100%) is a molecular ion (M^+^) and can be observed at *m/z* 396 for both compounds [48]. Small differences appear in the intensities of the fragment ion signals at *m/z* 381, where this ion is more intense for *cis*-CA and is formed due to the loss of a methyl group from the molecular ion. Further loss of the TMSO group produced the ion at *m/z* 293. Direct loss of the TMSO group from the molecular ion provides an [M−89]^+^ peak at *m/z* 307. The relatively intense fragment ion can be observed at *m/z* 219. The difference in mass 88 u, between *m/z* 307 and *m/z* 219 ions suggests further simultaneous elimination of the TMS and –CH_3_ groups from the [M−89]^+^ peak [49]. The ion at *m/z* = 73 is the trimethylsilyl moiety and is characteristic of TMS derivatives. The similar fragmentation of the CA TMS-derivative was observed in the studies reported by other authors [50,51]. The proposed fragmentation pathway for the TMS derivative is presented in Figure 2.

### 2.3. Stability of Trans-Ferulic Acid

The process of isomerization/degradation of *trans*-FA is similar to that of *trans*-CA, but in some cases even more rigorous. The results showed that the decrease in *trans*-FA content dissolved in MeOH or THF and exposed to different stress conditions is reflected in the increase in the *cis*-FA amount. Markedly promoted isomerization was again detected in MeOH solutions exposed to RT and daylight for 30 days (up to 43%). Similarly, in the study by Flores et al., a significant effect of sunlight on *trans*-FA degradation from western olive oil industry water was demonstrated [52]. On the other hand, the lowest degree of isomerization (5.2%) was observed when the solution of FA in THF was stored in the freezer at −18 °C for 1 day. Again, as described above for *trans*-CA, in the experiments with *trans*-FA dissolved in THF or MeOH and stored in the dark or in a refrigerator at −18 °C, a reversible reaction from the *trans* to *cis* form and vice versa was observed [46,47]. *Cis*-FA was the only degradation product of *trans*-FA identified by GC-MS. Chromatograms of silylated compounds present in an FA solution stored for 30 days in the freezer at −18 °C or at RT (darkness or daylight) are shown in Figure S3 (Supplementary Materials). The results of all stability tests performed on *trans*-FA dissolved in MeOH or THF are shown in Table 2.

#### Analysis and Identification of Ferulic Acid Isomers

**Figure 3:**
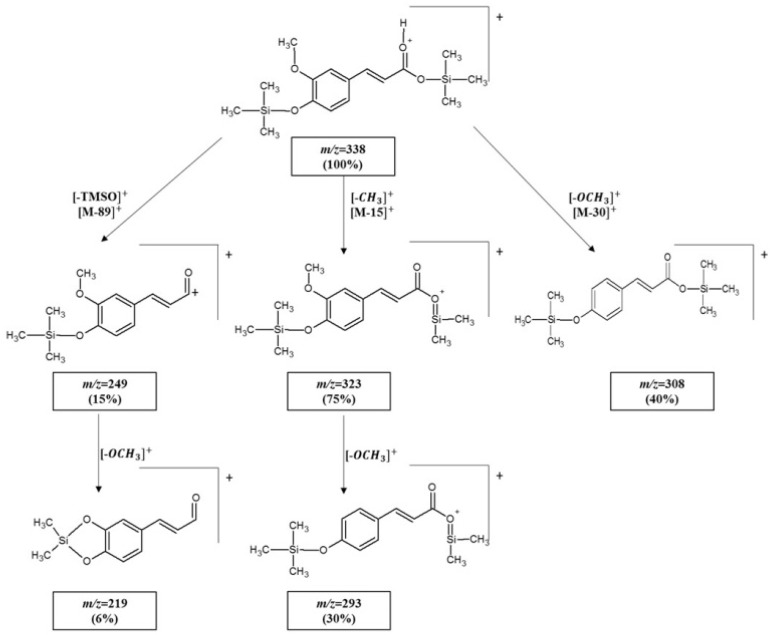
Proposed fragmentation pathway of the *trans*-FA-2-TMS derivative obtained under positive EI^+^ conditions.

*Cis*-FA and *trans*-FA are geometric isomers with very similar structures but can be well distinguished by the order of elution during gas chromatography (*trans*-FA is retained about 1 min longer than *cis*-FA) (Figure S3) and by the intensities of some fragment ion signals in their similar mass spectra (Figure S4, Supplementary Materials). Differences include the intensities of fragment ion signals at *m/z* 323, *m/z* 308, *m/z* 293 and *m/z* 249, where they are more intense for *cis*-FA than for *trans*-FA. A molecular M^+•^ peak is observed at *m/z* 338 and is the highest peak in both spectra. The loss of a methyl group from the molecular ion resulted in the fragment ion *m/z* 323, and further loss of the methoxy (-O-CH_3_) group produced the ion at *m/z* 293. Loss of the methoxy group from the main molecule led to the formation of the *m/z* 308 ion. Loss of the TMSO group from the molecular ion provided the *m/z* 249 ion, and further elimination of the -OCH_3_ group led to the fragment ion at *m/z* 219. The ion at *m/z* = 73 is the trimethylsilyl moiety and is characteristic of TMS derivatives [49]. The proposed scheme for the fragmentation pathway of derivatized *trans*-FA is presented in Figure 3.

### 2.4. Stability of Trans-Caffeic Acid and Trans-Ferulic Acid under the Influence of UV Radiation

In addition, short term stability tests of *trans*-CA and *trans*-FA dissolved in organic solvents (MeOH and THF) and exposed to UV radiation at two different wavelengths (254 nm and 366 nm) were carried out. From these results (Table 3), it can be concluded that *cis*-CA and *cis*-FA (31.1% and 33.6%, respectively) were formed in the largest quantities after exposure of the THF solutions to UV radiation at 366 nm for 6 h. The isomerization took place in both solvents at 366 nm to a greater extent than at 254 nm. This result indicated that the isomerization of *trans*-PAs seemed to be influenced by the corresponding absorption bands, i.e., for CA the absorption spectra showed the maximum absorption at 325 nm and for FA it showed the maximum absorption at 321 nm. The absorbance at 254 nm was minimal for both compounds [53,54]. Istasse et al. suggest that the isomerization of *trans*-hydroxycinnamic acids to their *cis*-forms can also occur at very low light exposure [35]. Light energy can unpair the electrons in the π bond so that free rotation around the C-C bond can take place. Consequently, after a 180° rotation, the unpaired electrons can pair up again, forming the other geometric isomer [8]. Horbur et al. also reported the strong UV absorption and potent antioxidant activity of CA and FA, which makes them promising sunscreen components [41].

From Table 1, Table 2 and Table 3, it is obvious that the concentration of *trans*-isomers in aged solutions not only decreased over time, but in some cases even increased. This can be explained by the fact that the isomerization of the investigated *trans*-isomers to their *cis*-forms is a reversible process that is dependent on solvent, temperature and time.

## 3. Materials and Methods

### 3.1. Chemicals

All reagents and solvents used were of analytical grade. Standards of *trans*-FA (99.5%) and *trans*-CA (99.5%), and solvents tetrahydrofuran (THF) and pyridine (99.9%) were purchased from Merck (Darmstadt, Germany). N-Methyl-N-(trimethylsilyl) trifluoroacetamide (MSTFA) was obtained from Sigma (St. Louis, MO, USA). HPLC-grade methanol (MeOH) was supplied from ChemLab (Zedelgem, Belgium), and toluene (99.5%) from Carlo Erba (Milano, Italy).

### 3.2. Preparation of Calibration Solutions and Calibration Curves

Stock solutions were prepared by accurately weighing 10 mg of the trans-CA and trans-FA standards, and dissolving them in 10 mL glass flasks with THF as a polar aprotic solvent or MeOH as a polar protic solvent, to obtain concentrations of 1000 mg L^−1^. Five calibration working solutions for both acids (concentration range from 1 to 100 mg L^−1^), in the two different solvents, were prepared from standard stock solutions by further dilution with the appropriate solvent. As the internal standard (ISTD) 2,5-dichlorobenzoic acid (2,5-DCBA) was used. The concentration of ISTD in all working solutions was 50 mg L^−1^.

The derivatization was carried out by evaporating the solvent to absolute dryness by rotary evaporation and by adding 100 μL of MSTFA and 50 μL of pyridine, and further by heating at 80 °C for 1 h in a sand bath. After derivatization was completed and the solutions cooled to room temperature, the solutions were transferred quantitatively to a 1 mL volumetric flask and diluted to the mark with toluene. The solutions were injected into the GC-MS system in triplicate. The calibration curves were constructed by linear regression of the peak area ratio of the individual trans-isomers to the ISTD (y) against the concentration in mg L^−1^ (x). The concentrations of the degradation products cis-CA and cis-FA, were quantified using trans-isomers, as their pure standards were not commercially available. In addition, three repeat analyses of the calibration solutions were performed within 2 weeks.

### 3.3. Instrumentation and GC-MS Conditions

Analyses were carried out on a Varian 3900 GC, coupled to an Varian Saturn 2100T MS. GC separation was performed using capillary column HP-5MS (30 m × 0.25 mm, with 5% phenyl methylpolysiloxane stationary phase 0.25 μm). A total of 1 µL of the sample was injected in the split injection mode (split ratio 1:10). The carrier gas was He (5.0 UHP) at 1.0 mL min^−1^ flow rate. The initial oven temperature was 40 °C, held for 1 min, and then the temperature was raised to 320 °C at a rate of 10 °C min^−1^ and finally held for 3 min. The total run time was 32 min. The injection-port was set to 250 °C and the transfer-line to 170 °C. A mass spectrometer recorded the entire spectrum (SCAN mode) in a range from *m/z* 50 to *m/z* 650, using electronic ionization energy at 70 eV. The identification of CA and FA from the samples was established by comparing their retention times and mass spectra with the derivatised standard compounds, while other compounds were identified by interpretations of their fragmentation patterns, by comparing their spectral properties with those reported in the literature or in the NIST mass spectra library.

### 3.4. Method Validation

The method was validated for linearity, precision as repeatability, limit of detection (LOD) and limit of quantification (LOQ). The repeatability of the analyses was evaluated using the relative standard deviation (% RSD) of three repeat analyses of five calibration solutions within one day. The repeatability of sample preparation (method precision) was determined by performing the complete analysis three times. The LOD and LOQ were determined to be the lowest injected concentration giving signal-to-noise ratios (S/N) of ≥ 3 and 10, respectively.

### 3.5. Stability Tests of Trans-Caffeic Acid and Trans-Ferulic Acid

The pure standards of *trans*-CA and *trans*-FA were dissolved in THF and MeOH to obtain concentrations of 1000 mg L^−1^. From each solution an aliquot of 100 μL was taken, 50 μL ISTD (γ = 1000 mg L^−1^) was added, evaporated, derivatized, diluted and measured by GC-MS immediately after preparation. To perform short and long-term stability studies, each solution was divided into a number of equal parts and transferred to graduate glass-stoppered test tubes. Four parts of each solution were placed in conditions of room temperature (RT) and daylight; the second four parts were placed in conditions of RT and darkness, and the last four parts were stored in the freezer in darkness at −18 °C for varied periods of time (1, 10, 20 and 30 days).

In parallel, three parts of each solution were exposed to UV at two different wavelengths, 254 nm and 366 nm, for different periods of time (2, 4 and 6 h). A UV cabinet (Camag 4) was used for this purpose. After exposure under these conditions, an aliquot of 100 μL from each solution was combined with 50 μL of the ISTD, evaporated, derivatized, diluted and analysed according to the procedure described above. The concentrations of the compounds investigated were determined from the corresponding freshly prepared calibration curves. The concentrations of the degradation products *cis*-CA and *cis*-FA were quantified based on the difference between the initial and the residual concentration of the corresponding *trans*-isomers.

## 4. Conclusions

There is a constant need for the qualitative and quantitative analysis of phenolic acids (PAs) in the pharmaceutical, cosmetic and food industries, as well as in medicine, biology, organic chemistry and biochemical analysis, and in environmental studies. In nature, PAs are present mainly as *trans*-isomers, but their degradation can occur under various stress conditions. Isomerism is particularly important from a pharmacological point of view, since different isomers may have different biological activities and therapeutic potential for the treatment or prevention of various human diseases. Consequently, in this study *trans*-caffeic acid (*trans*-CA) and *trans*-ferulic acid (*trans*-FA) were systematically tested and evaluated from the stability point of view after being disolved in two different solvents (methanol and tetrahydrofuran) and exposed to many different factors. The results obtained proved that the isomerization of *trans*-CA and *trans*-FA is a process that is dependent on the solvent, temperature and time. In general, aprotic solvents (THF), higher temperatures (room temperature) and longer exposure times (one month) led to the greater isomerization of both acids. *Cis*- isomers of the compounds studied were identified as the only degradation products in all tests. Geometrical isomers were successfully separated by gas chromatography (GC) and confirmed by mass spectrometry (MS) throughout the fragmentation patterns in their mass spectra. The described method is linear, repeatable (RSD < 3%) and precise. Our results have shown that GC-MS can be a challenge for the analysis of structurally related *cis-trans* forms of PAs and that it is also a suitable method for their determination in a very wide range of complex natural matrices.

## Figures and Tables

**Table 1:** Isomerization of trans-CA dissolved in THF or MeOH under the influence of various storage conditions.

Solvent	Storage Conditions (Day/Days)	RT				Freezing at −18 °C	
		Darkness		Daylight			
		*Trans (%)*	*Cis (%)*	*Trans (%)*	*Cis (%)*	*Trans (%)*	*Cis (%)*
THF	1	96.2 ± 4.2	3.8 ± 0.1	75.5 ± 2.1	24.5 ± 0.2	96.8 ± 2.4	3.2 ± 0.1
	10	85.1 ± 3.8	14.9 ± 0.7	70.9 ± 0.5	29.1 ± 0.5	85.7 ± 5.3	14.3 ± 0.2
	20	90.8 ± 5.4	9.2 ± 0.6	69.5 ± 1.7	30.5 ± 0.5	92.5 ± 3.0	7.5 ± 0.1
	30	91.8 ± 5.6	8.2 ± 0.1	67.4 ± 1.4	32.6 ± 0.1	92.1 ± 0.7	7.9 ± 0.9
MeOH	1	95.3 ± 3.7	4.7 ± 0.1	72.1 ± 1.5	27.8 ± 0.4	94.9 ± 2.1	5.1 ± 0.1
	10	79.8 ± 2.2	20.2 ± 0.3	68.4 ± 1.4	31.6 ± 0.5	86.1 ± 2.6	13.9 ± 0.1
	20	82.5 ± 1.9	17.5 ± 0.2	65.7 ± 1.1	34.3 ± 0.5	86.4 ± 1.8	13.6 ± 0.7
	30	82.6 ± 2.0	17.5 ± 0.2	65.4 ± 2.0	34.6 ± 0.7	87.1 ± 1.2	12.9 ± 0.4

**Table 2:** Isomerization of *trans*-FA dissolved in THF or MeOH under the influence of different storage conditions.

Solvent	Storage Conditions (Day/Days)	RT				Freezing at −18 °C	
		Darkness		Daylight			
		*Trans (%)*	*Cis (%)*	*Trans (%)*	*Cis (%)*	*Trans (%)*	*Cis (%)*
THF	1	92.8 ± 2.8	7.3 ± 0.1	74.6 ± 1.0	25.4 ± 0.2	94.8 ± 1.3	5.2 ± 0.2
	10	79.3 ± 4.3	20.7 ± 0.5	66.7 ± 0.6	33.3 ± 0.6	78.9 ± 4.2	21.1 ± 0.4
	20	88.5 ± 5.8	11.5 ± 0.2	66.9 ± 2.1	33.1 ± 1.6	88.5 ± 3.4	11.5 ± 0.3
	30	80.3 ± 1.8	19.7 ± 0.4	63.4 ± 2.0	36.6 ± 0.6	80.7 ± 1.2	19.3 ± 0.9
MeOH	1	94.7 ± 1.7	5.3 ± 0.4	57.5 ± 2.1	42.5 ± 1.1	89.3 ± 2.9	10.7 ± 0.3
	10	79.5 ± 1.9	20.5 ± 0.3	60.4 ± 1.0	39.6 ± 0.3	78.4 ± 0.4	21.6 ± 0.4
	20	80.9 ± 2.0	19.1 ± 1.0	58.9 ± 0.7	41.1 ± 0.8	83.9 ± 4.5	16.1 ± 1.9
	30	73.6 ± 1.7	26.1 ± 2.0	57.9 ± 2.3	42.8 ± 0.9	79.2 ± 0.4	20.8 ± 0.2

**Table 3:** Isomerization of *trans*-CA and *trans*-FA dissolved in THF or MeOH under the influence of UV radiation at two wavelengths (254 nm and 366 nm).

Solvent	Wavelength (nm)	Exposure Time (h)	CA		FA	
			*Trans (%)*	*Cis (%)*	*Trans (%)*	*Cis (%)*
THF	254	2	96.3 ± 1.2	3.9 ± 1.2	94.2 ± 2.1	6.5 ± 1.0
		4	93.8 ± 0.2	6.3 ± 0.3	95.2 ± 1.2	4.4 ± 0.6
		6	90.8 ± 1.0	9.2 ± 1.1	98.7 ± 1.3	1.3 ± 0.9
	366	2	85.5 ± 1.7	14.5 ± 1.7	75.2 ± 0.7	24.8 ± 1.0
		4	82.3 ± 0.9	17.7 ± 1.0	74.1 ± 1.5	25.9 ± 0.8
		6	87.4 ± 1.6	12.6 ± 1.5	71.3 ± 2.3	28.7 ± 2.1
MeOH	254	2	94.9 ± 3.1	5.1 ± 3.0	90.6 ± 1.2	9.3 ± 1.1
		4	87.5 ± 0.4	12.5 ± 0.6	86.8 ± 1.0	13.2 ± 0.8
		6	74.7 ± 0.5	25.3 ± 0.4	73.0 ± 0.7	27.0 ± 0.5
	366	2	79.6 ± 1.1	20.3 ± 1.0	74.1 ± 2.4	25.9 ± 1.6
		4	73.2 ± 2.7	26.8 ± 1.6	69.4 ± 3.8	30.6 ± 3.2
		6	68.9 ± 0.8	31.1 ± 0.7	66.4 ± 1.3	33.6 ± 1.6

## Data Availability

The authors confirm that the data supporting the findings of this study are available within the article and its Supplementary Materials.

## Supplementary Materials

Figure S1: Typical chromatogram of silylated compounds in a freshly prepared solution of *trans*-CA in MeOH, Figure S2: EI mass spectra of: (a) *trans*-CA-3-TMS derivative; (b) *cis*-CA-3-TMS derivative, Figure S3: Typical chromatograms of silylated compounds present in a FA-THF-solution after 30 days stored: (a) in the freezer at −18 °C; (b) at RT and darkness (c) at RT and daylight, Figure S4: EI mass spectra of: (a) *trans*-FA-3-TMS derivative; (b) *cis*-isomer.
