# Supplementary material for: Validated Stability-Indicating GC-MS Method for Characterization of Forced Degradation Products of Trans-Caffeic Acid and Trans-Ferulic Acid
Source: Molecules. 2021 Apr 23;26(9):2475. doi: 10.3390/molecules26092475 (PMC8123059; doi:10.3390/molecules26092475)
Supplement: Supplementary file 1 [file molecules-26-02475-s001.zip › molecules-1176802-supplementary.pdf]

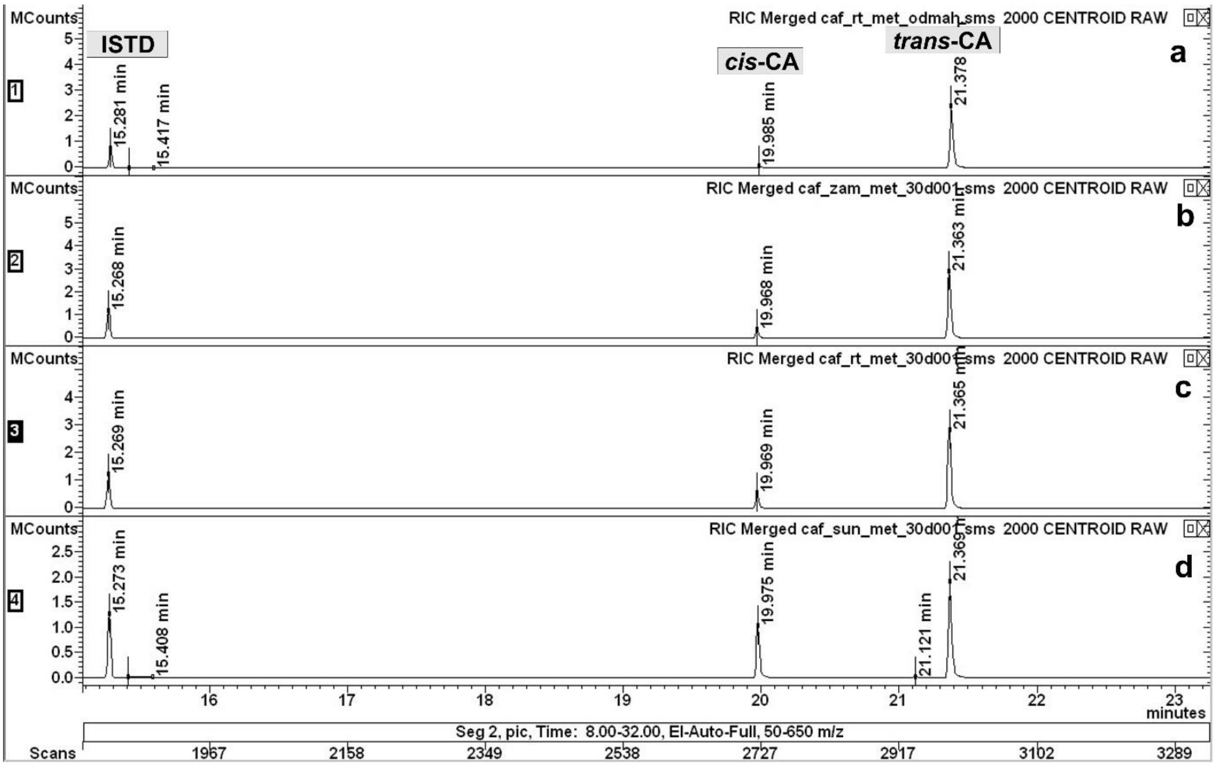

Figure S1. Typical chromatograms of silylated compounds present in a CA-MeOH-solution: a) freshly prepared b) after 30 days stored in the freezer at -18 °C; c) after 30 days stored at RT and darkness d) after 30 days stored at RT and daylight.

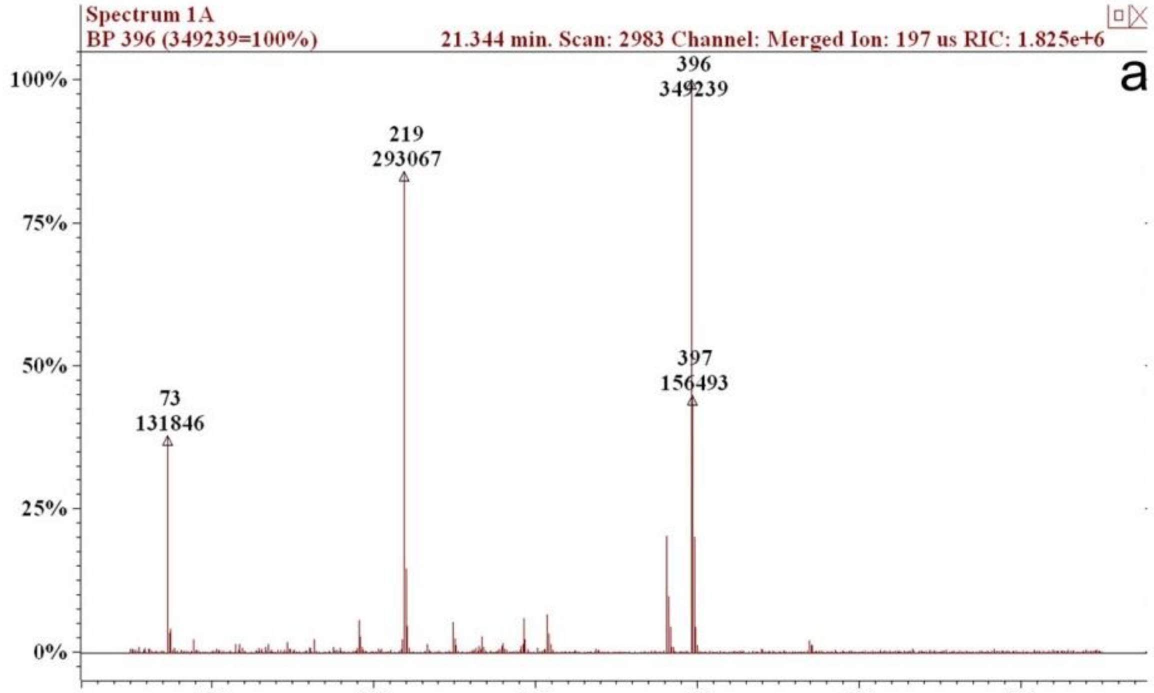

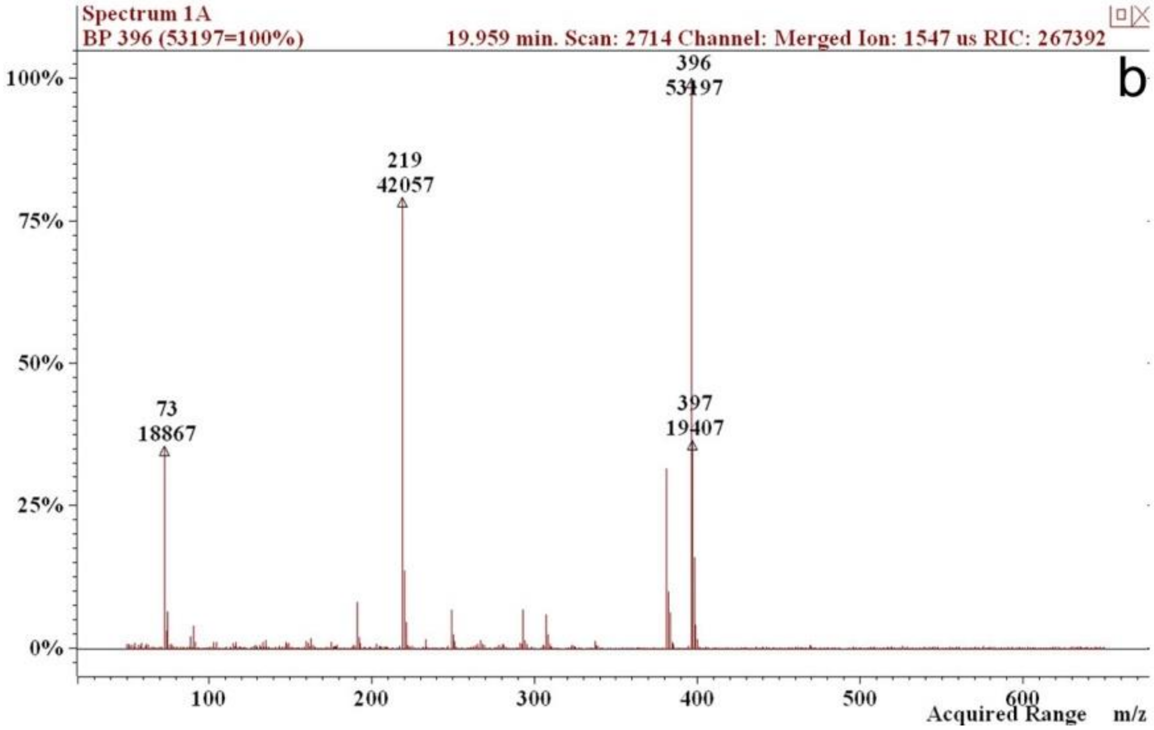

Figure S2. EI mass spectra of: a) *trans*-CA-3-TMS derivative; b) *cis*-CA-3-TMS derivative.

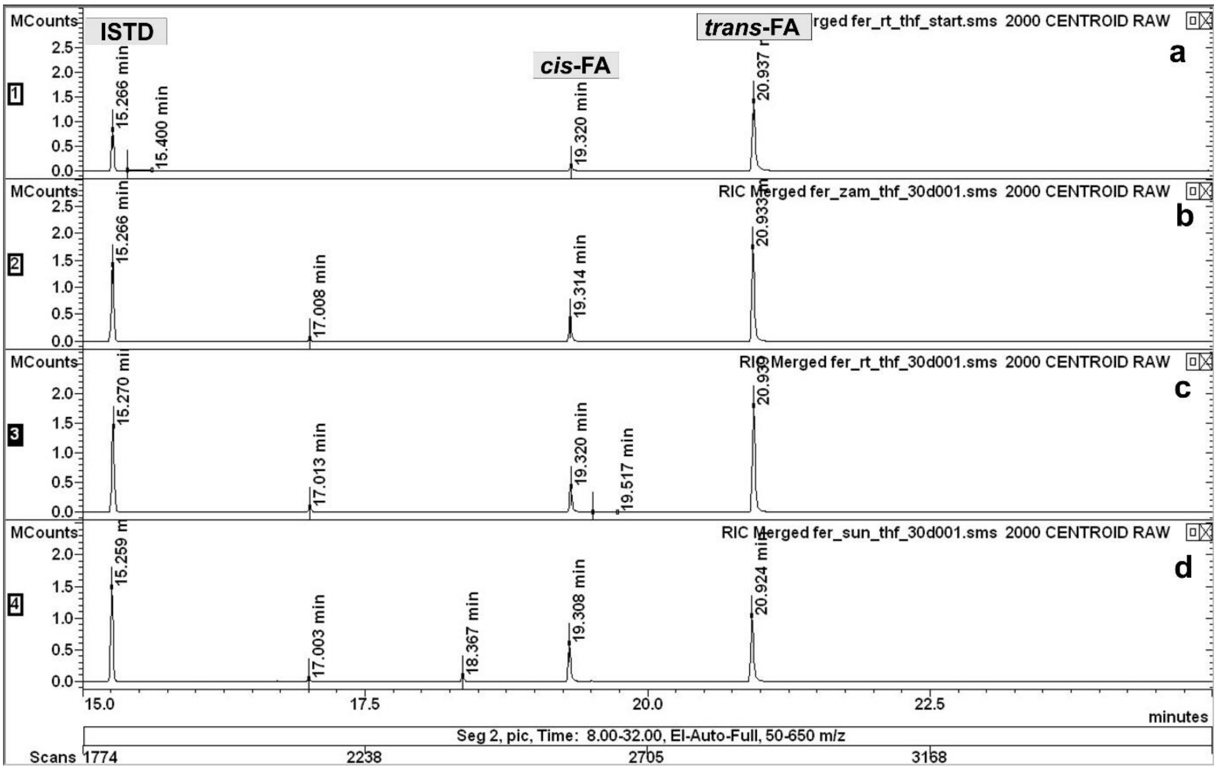

Figure S3. Typical chromatograms of silylated compounds present in a FA-THF-solution: a) freshly prepared b) after 30 days stored in the freezer at -18 °C; c) after 30 days stored at RT and darkness d) after 30 days stored at RT and daylight.

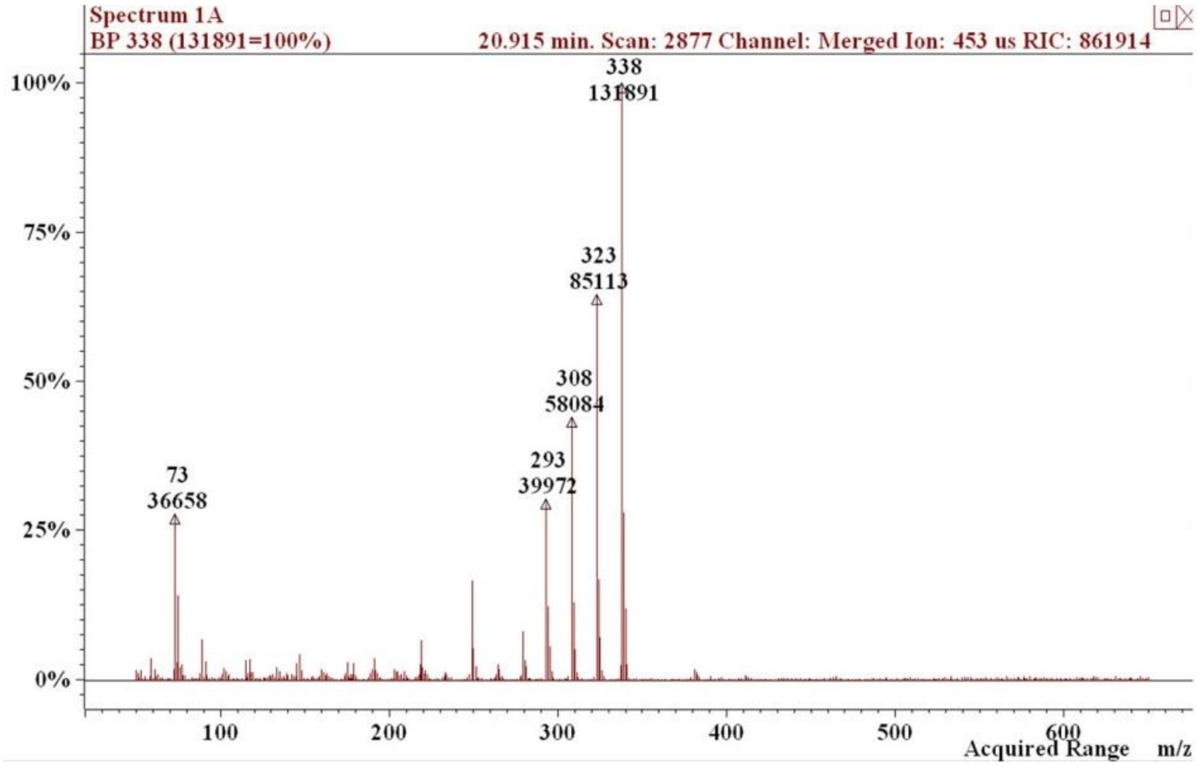

Spectrum 1A
BP 338 (131891=100%)
20.915 min. Scan: 2877 Channel: Merged Ion: 453 us RIC: 861914
338
131891
323
85113
308
58084
293
39972
73
36658
100%
75%
50%
25%
0%
100
200
300
400
500
600
Acquired Range
m/z

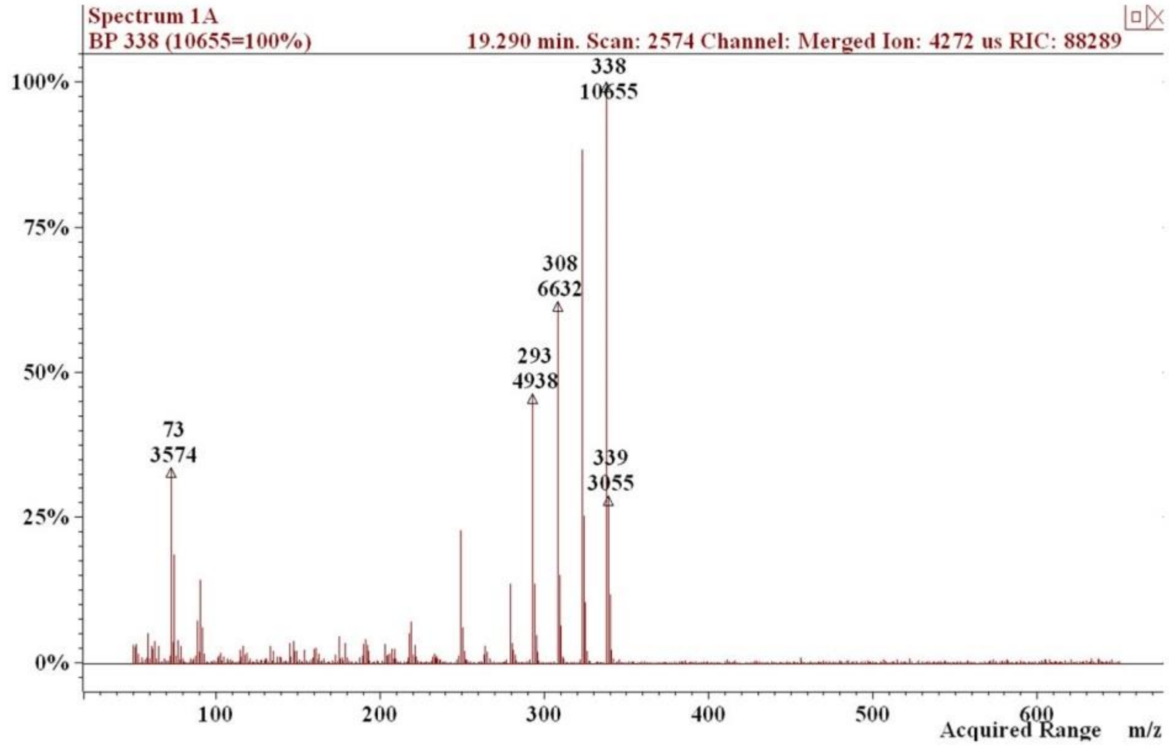

Figure S4. EI mass spectra of: a) *trans*-FA-3-TMS derivative; b) *cis*-isomer.
